# Supplementary material for: Integrative analysis of epigenetic subtypes in acute myeloid Leukemia: A multi-center study combining machine learning for prognostic and therapeutic insights
Source: PLoS One. 2025 May 28;20(5):e0324380. doi: 10.1371/journal.pone.0324380 (PMC12118855; doi:10.1371/journal.pone.0324380)
Supplement: Attachment — Submitted filename: Plosone review.docx [file pone.0324380.s001.docx]

1. The manuscript uses multiple data sources, but it is not always clear how batch effects were corrected across datasets. Suggest adding a table summarizing datasets, sample sizes, key molecular features analyzed, and preprocessing steps.
2. While the study identifies 20 key epigenetic features, the rationale for their selection is unclear. The authors mention empirical Bayesian-based clustering but do not explicitly explain how these features were prioritized over others. Authors should provide a detailed explanation of the criteria used to define epigenetic relevance.
3. The Random Survival Forest (RSF) model is applied and validated across multiple cohorts, but there is no comparison with simpler or commonly used models such as Cox proportional hazards or LASSO regression. Authors should consider adding a comparison with conventional models to demonstrate the added value of RSF. A sensitivity analysis showing the impact of different hyperparameters on model performance would strengthen reproducibility.
4. While the study integrates multiple datasets, there is no independent prospective validation in a clinical setting.
5. The drug sensitivity analysis identifies epigenetic regulators (LAQ824, MS-275) and proteasome inhibitors (Bortezomib, MG-132) as differentially effective in risk groups. However, these findings are based on in silico predictions rather than experimental validation. A discussion on potential in vitro or in vivo validation strategies would be valuable.
6. Some figures (e.g., Kaplan-Meier curves) would benefit from additional statistical annotations such as hazard ratios (HR) with confidence intervals.
7. Ensure consistent terminology across the manuscript (e.g., AML subtypes are sometimes referred to as CS1/CS2 and sometimes as high-/low-risk).
8. Clearly define abbreviations at first mention.
9. The manuscript states "Ethics approval and consent to participate: NA"—is this accurate given the use of patient-derived datasets? If datasets were de-identified, state that explicitly.
