# Supplementary material for: Integrative analysis of epigenetic subtypes in acute myeloid Leukemia: A multi-center study combining machine learning for prognostic and therapeutic insights
Source: PLoS One. 2025 May 28;20(5):e0324380. doi: 10.1371/journal.pone.0324380 (PMC12118855; doi:10.1371/journal.pone.0324380)
Supplement: Attachment — Submitted filename: Response to Reviewers AML.docx [file pone.0324380.s003.docx]

**Reviewer 1:**

1. **The manuscript uses multiple data sources, but it is not always clear how batch effects were corrected across datasets. Suggest adding a table summarizing datasets, sample sizes, key molecular features analyzed, and preprocessing steps.**

We appreciate the reviewer's suggestion. We have added a comprehensive table (Table 1) summarizing all datasets used, their sample sizes, sources, and key molecular features analyzed. The preprocessing steps and how to correct for batch effects are described in the “TCGA-LAML Data Preprocessing and Integration” and “Multi-Dataset Expression Profile Integration and Batch Effect Correction” sections of the Methods section. Specifically, our approach included:

1. **Standardized preprocessing pipeline**: For the TCGA-LAML dataset, we implemented systematic preprocessing by normalizing TPM values for protein-coding genes, lncRNAs, and miRNAs, with log2 transformation to achieve approximate normal distribution. DNA methylation data retained beta values from the Illumina 450K platform.
2. **Rigorous sample quality control**: We developed specific sample screening functions to retain only primary hematological tumor samples and ensured sample consistency across all omics data through ID matching.
3. **Integration of cross-platform data**: Our multi-dataset integration involved retaining only genes common to all datasets and samples with complete survival information.
4. **Batch effect correction**: We employed the ComBat algorithm using an empirical Bayesian framework to effectively remove non-biological variation between different datasets. This approach is widely recognized for harmonizing gene expression data from different sources.
5. **Quality assessment**: We used Principal Component Analysis (PCA) to assess the effectiveness of batch correction before proceeding with downstream analyses.
2. **While the study identifies 20 key epigenetic features, the rationale for their selection is unclear. The authors mention empirical Bayesian-based clustering but do not explicitly explain how these features were prioritized over others. Authors should provide a detailed explanation of the criteria used to define epigenetic relevance.**

We sincerely appreciate the reviewer's astute observation regarding the selection of 20 key epigenetic features. We acknowledge that the methodological details concerning this selection process were not sufficiently elucidated in our manuscript and would like to provide a comprehensive explanation.

Our feature selection methodology followed a systematic multi-step process:

Initially, we applied empirical Bayesian-based consensus clustering analysis to the TCGA dataset, which successfully identified two molecular subtypes with significantly different survival outcomes. This initial classification established the foundation for subsequent analyses.

Subsequently, we conducted differential expression analysis between these two prognostically distinct subtypes to identify candidate genes potentially influencing patient outcomes. This step was predicated on the hypothesis that genes exhibiting significant differences between prognostic subtypes likely play crucial roles in disease progression and epigenetic regulation.

We then evaluated these candidate features across multiple predictive models, including traditional statistical approaches and machine learning methods (such as Cox proportional hazards models, support vector machines, and random forests). Through systematic comparison, the Random Survival Forest (RSF) model demonstrated superior predictive performance.

Within the RSF model, we ranked all features based on variable importance scores, which quantify each feature's contribution to predicting patient prognosis. We selected the top 20 features with the highest importance scores (including CPNE8, CD109, and CHRDL1), which exhibited the most significant impact on the predictive model.

Finally, to validate the robustness of the selected features, we performed cross-validation across multiple independent cohorts, including TCGA-LAML, BEAT, TARGET, GSE12417, and GSE37642. These features consistently demonstrated strong predictive capability across different cohorts, further confirming their importance and epigenetic relevance.

Through this systematic approach, we ensured that the 20 key epigenetic features selected not only possess statistical significance but also demonstrate strong biological relevance to disease progression and robust prognostic value across diverse patient populations.

3. **The Random Survival Forest (RSF) model is applied and validated across multiple cohorts, but there is no comparison with simpler or commonly used models such as Cox proportional hazards or LASSO regression. Authors should consider adding a comparison with conventional models to demonstrate the added value of RSF. A sensitivity analysis showing the impact of different hyperparameters on model performance would strengthen reproducibility.**

We appreciate the reviewer's important point. To clarify, as part of our rigorous model selection process, we conducted a comprehensive comparison between RSF and traditional models, including Cox proportional hazards and LASSO regression. As illustrated in Figure 4A of our manuscript, we evaluated 82 distinct machine learning algorithms, encompassing traditional statistical models such as Cox proportional hazards models, penalized regression models (LASSO, Ridge, and Elastic Net), and various ensemble learning methods. The heatmap in Figure 4A provides a comprehensive comparison of C-indices across all models and validation datasets, with color intensity representing predictive performance (C-index values ranging from 0-1).

Our comparative analysis demonstrated that the RSF model consistently outperformed traditional models in terms of C-index across multiple validation cohorts. This comprehensive comparison substantiates the added value of utilizing the RSF model for our specific dataset and research question. The superior performance of RSF can be attributed to its ability to handle non-linear relationships and complex interactions among variables without making assumptions about the underlying data distribution, which is particularly valuable in heterogeneous diseases such as AML.

Regarding hyperparameter optimization, we conducted extensive sensitivity analyses during model development, employing 5-fold cross-validation to determine optimal hyperparameter settings. For the RSF model, we tested various combinations of the following:

- Number of trees (500, 1000, 1500, 2000)
- Minimum node size (1, 3, 5, 10)
- Number of variables tried at each split (3, 5, 7, 9, sqrt(p), p/3)
- Splitting rules ('logrank', 'extratrees', 'C', 'maxstat')

The final model parameters were selected based on the highest cross-validation C-index. We found that a configuration using the 'logrank' splitting rule with sqrt(p) variables tried at each split provided the most robust performance across validation cohorts.

4. **While the study integrates multiple datasets, there is no independent prospective validation in a clinical setting.**

We greatly appreciate your insightful comments. We have discussed the limitation of the lack of prospective experiments in the "Limitations" section and are committed to conducting prospective clinical studies in the near future. Meanwhile, we have incorporated single-cell analysis into the manuscript, which plays a crucial role in bridging this gap. By focusing on individual cells, we were able to validate the biological significance of the RSF risk score model with high precision. For example, we successfully distinguished between malignant and non-malignant hematopoietic stem cells (HSC) with a sensitivity as high as 82.70%, outperforming the existing LSC17 scoring system. Moreover, our single-cell analysis revealed key evidence, such as a significant increase in epigenetic scores in malignant HSC, which directly confirms the central role of epigenetic regulation in the pathogenesis of AML, a revelation that cannot be obtained through traditional analyses:

"**Single-cell Transcriptome Analysis to Validate the Biological Significance of the Model**

To explore and validate the biological significance of the RSF risk score model at the single-cell level, this study analyzed single-cell RNA sequencing data from AML patients. The phs000159 dataset was obtained from the ABC portal database (http://abc.sklehabc.com/). This dataset was first generated by Allegra A. Petti et al. in 2019 using the 10X Genomics platform and published in Nature Communications. It contains single-cell sequencing results of 87,538 bone marrow samples from AML patients.

After importing the pre-processed matrix to construct a Seurat object, we integrated the pre-calculated UMAP dimensionality reduction coordinates and cell type annotation information to build a complete single-cell analysis framework. To distinguish between malignant and non-malignant cells, the cell malignancy status annotation information was imported and integrated into the analysis process. Particularly, this study focused on the hematopoietic stem cell (HSC) sub-population, which is of utmost importance in the hematopoietic system. This key cell population was identified through cell-type markers, and malignant HSC and non-malignant HSC were further distinguished.

To evaluate the role of epigenetic regulation in the pathogenesis of AML, this study utilized the epigenetic-related gene set organized above and calculated the epigenetic regulation activity score for each HSC cell using the ssGSEA method. Subsequently, the characteristics of the spatial distribution of these scores in cells were visualized by UMAP, and the differences in epigenetic activities between malignant and non-malignant HSC were compared using the Wilcoxon rank-sum test.

Most importantly, using the same ssGSEA method, this study calculated the LSC17 score and the expression score of high-weight genes in the RSF model for each HSC cell, respectively. LSC17 is a widely validated gene set related to AML stem cells, containing 17 genes closely related to the functions of leukemia stem cells. The RSF model score is based on the 20 genes with the highest weights in the newly developed random forest model of this study. These two sets of scores represent classic and newly developed prognostic markers of AML, respectively. This study analyzed again using the same statistical scheme and quantitatively compared the difference patterns between malignant and non-malignant HSC.

**Single-cell Analysis in HSC of AML**

To validate and further explore the biological significance of the RSF risk score model at the single-cell level, this study analyzed single-cell RNA sequencing data of AML patients obtained from the ABC portal website. UMAP visualization of the entire single-cell dataset revealed distinct clustering patterns of different cell types in the AML bone marrow microenvironment (Figure 11A). Cell type annotation identified multiple cell populations, including HSC, myeloid progenitor cells, monocytes, lymphocytes, and erythroid precursor cells, highlighting the complex cell composition in AML samples.

When cells were classified according to their malignant status (Figure 11B), malignant cells (red) and non-malignant cells (blue) showed differential distributions in the UMAP space. Malignant cells formed several distinct clusters, which may represent different leukemia clones or differentiation states. Given the crucial role of HSC in the pathogenesis of AML and its highest cell number and proportion, this study specifically extracted the HSC population for in-depth analysis. Notably, this study first used epigenetic regulation score calculation to reveal the UMAP visualization of the activity of epigenetic programs between malignant and non-malignant HSC (Figure 11C). It showed that malignant HSC (mainly in the red area) had significantly higher epigenetic scores compared to non-malignant HSC (mainly in the blue area), indicating that abnormal epigenetic regulation is a key feature of leukemia stem cells.

Statistical comparison confirmed that the epigenetic scores of malignant HSC were significantly higher than those of non-malignant HSC (P < 0.0001, Figure 11D). This finding is consistent with the premise of this study and the overall RNA-seq observations, supporting the crucial role of epigenetic reprogramming in the pathogenesis of AML.

To validate the clinical relevance of the prognostic model of this study at the single-cell level, this study continued to calculate the LSC17 score and the RSF model score in HSC respectively. The LSC17 score, based on 17 leukemia stem cell-characteristic genes, was significantly higher in malignant HSC than in non-malignant HSC (P < 0.0001, Figure 11E), confirming its ability to detect leukemia stem cell characteristics at the single-cell level. Most importantly, the RSF model score based on the top 20 prognostic genes also showed a significant distinction between malignant and non-malignant HSC (P < 0.0001, Figure 11F). The significant increase in the RSF score in malignant HSC provided convincing evidence that the prognostic model of this study captured the fundamental biological characteristics of leukemia stem cells, which may explain its robust performance in predicting patient prognosis across multiple cohorts.

To further evaluate the performance of epigenetic scores, LSC17 scores, and RSF scores in distinguishing malignant from non-malignant HSC, this study divided cells into high- and low- expression groups based on the median of each score, and constructed contingency tables and performance index comparisons (Tables 2 and 3). Table 2 shows the association between the grouping based on different scoring methods and the malignant status of cells. All three scoring methods showed a higher proportion of high-score groups in malignant HSC, while low-score groups were dominant in non-malignant HSC. The RSF score identified 2,060 high-score cells in malignant cells (accounting for approximately 82.70% of the total number of malignant cells), and correctly classified 12,356 low-score cells in non-malignant cells (accounting for approximately 53.53% of the total number of non-malignant cells). Table 3 quantitatively compared the diagnostic performance of the three scoring methods. The results showed that the RSF score outperformed the epigenetic score and the LSC17 score in multiple indicators such as sensitivity (82.70%), specificity (53.53%), precision (16.11%), F1- score (26.98%), accuracy (56.59%), and negative predictive value (96.63%), indicating that the RSF score model not only has excellent prognostic prediction ability at the overall cohort level but also has high discriminative efficacy in distinguishing malignant from non-malignant HSC at the single-cell level. These findings further validate the ability of the RSF model to capture the biological characteristics of AML from the perspective of single-cell resolution, providing a more solid theoretical basis for its clinical application.

**Conclusion**

Moreover, compared with traditional whole-tissue-based analyses, the single-cell RNA sequencing analysis in this study provides an unprecedented perspective on cellular heterogeneity. Existing AML studies are mostly based on whole-tissue samples, which are unable to distinguish the contributions of malignant and non-malignant cells, and it is also difficult to capture the characteristics of key cell subsets. Although the single-cell studies by Wu et al. and Ediriwickrema et al. described the cellular heterogeneity of AML, they failed to integrate epigenetic regulation with prognostic prediction models. This study is the first to validate the biological basis of the RSF prognostic model at the single-cell level, demonstrating that it can effectively distinguish malignant from non-malignant HSC with a sensitivity of 82.70%, outperforming the established LSC17 scoring system. Notably, the single-cell analysis in this study revealed a significant increase in epigenetic scores in malignant HSC, providing direct evidence for the conclusion drawn from the multi-omics level in this study that "epigenetic regulation plays a central role in the pathogenesis of AML", which cannot be achieved by traditional whole-tissue analyses. "

5. **The drug sensitivity analysis identifies epigenetic regulators (LAQ824, MS-275) and proteasome inhibitors (Bortezomib, MG-132) as differentially effective in risk groups. However, these findings are based on in silico predictions rather than experimental validation. A discussion on potential in vitro or in vivo validation strategies would be valuable.**

Thank you for your suggestion. In vitro, we can use AML cell lines with well - defined molecular subtypes and risk scores to test the efficacy of these drugs. For example, treat high - risk and low - risk cell lines with LAQ824, MS - 275, Bortezomib, and MG - 132 respectively, and then evaluate cell viability, proliferation, and apoptosis. In vivo, AML mouse models can be established by xenografting human AML cells or by genetically engineering mice to develop leukemia. These models will enable us to assess the real - world efficacy of these drugs in different risk - group contexts, taking into account factors such as drug pharmacokinetics, immune - tumor interactions, and tumor microenvironment effects. This experimental validation can not only confirm our in - silico predictions but also provide more reliable evidence for clinical drug selection and treatment optimization. We have added the relevant elaboration to the manuscript: “Furthermore, the lack of in vitro and in vivo functional experimental validation limits causal relationship determinations.”“(5) Validating drug sensitivity findings experimentally. For example, we should test drugs like LAQ824, MS-275, Bortezomib, and MG-132 on AML cell lines (in vitro) and mouse models (in vivo)”

6. **Some figures (e.g., Kaplan-Meier curves) would benefit from additional statistical annotations such as hazard ratios (HR) with confidence intervals.**

We truly appreciate your suggestion. In response to your comment, we have carefully reviewed all relevant figures, especially those like the Kaplan-Meier curves. We have now added the necessary statistical annotations, including HR with confidence intervals, as you recommended. We have highlighted these added annotations in yellow within the text.

7. **Ensure consistent terminology across the manuscript (e.g., AML subtypes are sometimes referred to as CS1/CS2 and sometimes as high-/low-risk).**

We appreciate the reviewer's attention to terminological consistency throughout our manuscript. We would like to clarify the distinct entities referenced by these different terms in our study.

In our initial multi-omics integrative analysis of the TCGA-LAML cohort, we identified two distinct AML subtypes, designated as CS1 and CS2. These subtypes are characterized by significant differences in molecular features at the transcriptomic, epigenomic, and genomic levels. Notably, CS2 patients demonstrated significantly superior overall survival compared to CS1 patients (P < 0.001).

Following this classification, we developed a Random Survival Forest (RSF) model to create a more clinically relevant prognostic tool. This model incorporated various molecular features and the LSC17 score. Based on the RSF model's variable importance analysis and optimal cutpoint methodology, patients were then stratified into high-risk and low-risk groups.

It is important to emphasize that CS1/CS2 and high-risk/low-risk refer to different classification systems within our study. The CS1/CS2 subtypes represent the primary molecular clusters identified through empirical Bayesian-based consensus clustering of multi-omic data. In contrast, the high-risk and low-risk designations represent the prognostic stratification derived from our machine learning model using differentially expressed genes between these initial clusters.

The key epigenetic features identified from the RSF model, such as CPNE8, CD109, and CHRDL1, were used to calculate a risk score for each patient, which formed the basis for distinguishing between high-risk and low-risk groups.

We have thoroughly reviewed the manuscript to ensure consistent terminology throughout: CS1/CS2 is used exclusively when referring to the molecular subtypes identified through multi-omics clustering, while high-risk and low-risk designations are used consistently when discussing prognostic stratification and clinical outcomes prediction.

8. **Clearly define abbreviations at first mention.**

Thank you so much for your careful suggestion. We have carefully reviewed the manuscript and defined all abbreviations at their first mention. Each abbreviation now has a corresponding full - name explanation. For example, in the "Materials and Methods" section, "TCGA - LAML" is explained as "The Cancer Genome Atlas - Acute Myeloid Leukemia" upon its initial mention, and so on for other abbreviations such as "GDSC".

9. **The manuscript states "Ethics approval and consent to participate: NA"—is this accurate given the use of patient-derived datasets? If datasets were de-identified, state that explicitly.**

We appreciate you raising this important point. We have revised this section to more accurately reflect the ethical considerations of our study. The updated statement reads:

"Ethics approval and consent to participate: This study utilized publicly available, de-identified datasets from multiple repositories (TCGA-LAML, BEAT database, TARGET Project, and GEO datasets GSE12417 and GSE37642). All original data collection was conducted with appropriate patient consent and ethical approval by the respective institutions that generated these datasets. As our study involved only secondary analysis of de-identified, publicly available data, additional ethics approval was not required for our specific analyses according to institutional policies. All data usage complied with the data access policies and terms of service for each repository."

This revision clarifies that we used de-identified public datasets that were collected with appropriate consent in their original studies, which is consistent with common practices for secondary analyses of public genomic data repositories like TCGA, GEO, and TARGET.

**Reviewer 2:**

1. **The study mainly uses datasets (TCGA, BEAT, TARGET, etc.) from Western populations. The applicability of CS1 and CS2 subtypes to other ethnic groups (e.g., Asian, African) remains unclear. Validation in diverse populations is needed.**

We sincerely thank you for your concern regarding the applicability of our research findings to different ethnic groups. We acknowledge that this study mainly utilized datasets from Western populations, which indeed raises valid questions about the applicability of our identified CS1 and CS2 subtypes, as well as the high - and low - risk groups, to other ethnic groups such as Asians and Africans.

However, we adopted a multi - cohort integration approach, combining data from multiple independent datasets, with the aim of enhancing the statistical power and reliability of the results. The consistent patterns observed across these datasets, such as the significant survival differences between the high - and low - risk groups and the prognostic performance of our random survival forest model, suggest a certain degree of universality in the underlying molecular mechanisms.

Nonetheless, we fully recognize the importance of validating our research findings in diverse populations and are committed to addressing this issue through future research. We will conduct large - scale clinical validation studies in the future. We plan to collaborate with international research teams to collect data from AML patients of Asian, African, and other ethnic groups. Through data integration, we can directly assess the stability and applicability of the high - and low - risk groups across different ethnic groups.

We have added discussions related to ethnicity in the limitations section of the Discussion:” The third limitation is the ethnic diversity of the study population. The datasets used in this study mainly originated from Western populations. The genetic and epigenetic backgrounds of different ethnic groups can vary, which may affect the applicability of our risk groups.”

2. **CIBERSORT, ESTIMATE, and xCell are used to infer the immune microenvironment, but they lack direct validation with patient samples. Using flow cytometry (FACS) on AML patient samples would help confirm whether CS1 is enriched with Treg cells and M2 macrophages.**

We sincerely appreciate the reviewer's valuable suggestion regarding the validation of immune microenvironment inferences using flow cytometry. We acknowledge that direct experimental validation would significantly enhance the reliability of results derived from computational methods such as CIBERSORT, ESTIMATE, and xCell.

In the present study, our primary objective was to develop and validate a prognostic prediction model based on epigenetic features. Due to resource constraints and study design considerations, we prioritized computational biology approaches to predict the immune microenvironment characteristics in AML. This approach allowed us to conduct preliminary explorations across large-scale cohorts, providing direction for subsequent experimental validation.

Notably, we have already performed partial validation of our RSF model at the single-cell level. As demonstrated in Figure 11 of our manuscript, we analyzed single-cell RNA sequencing data from AML patients, with particular focus on the hematopoietic stem cell (HSC) population. Our single-cell analysis revealed that the RSF model effectively distinguishes between malignant and non-malignant HSCs with a sensitivity of 82.70%, outperforming the established LSC17 scoring system. This finding provides strong support for the biological significance of our model at the cellular heterogeneity level. Specifically, we observed significantly higher epigenetic scores in malignant HSCs compared to non-malignant HSCs, directly supporting our conclusion from multi-omics analysis that "epigenetic regulation plays a central role in the pathogenesis of AML."

Regarding the reviewer's suggestion, we fully agree that flow cytometry validation would substantially strengthen our findings. Therefore, we have incorporated the following elements into our future research plans:

1. **Flow Cytometry Validation of Immune Microenvironment**: We plan to conduct multi-parameter flow cytometry analysis on fresh AML patient samples, focusing on the proportions of Treg cells (CD4+CD25+FOXP3+) and M2 macrophages (CD68+CD163+). We will compare these cell populations between CS1 and CS2 subtype patients to directly validate the predictions from computational methods.
2. **Functional Validation Experiments**: We intend to investigate the interactions between Treg cells, M2 macrophages, and AML malignant clones through in vitro co-culture experiments, assessing how these immunosuppressive cells influence AML cell proliferation, apoptosis, and drug resistance.
3. **Development of Clinical Detection Methods for Epigenetic Markers**: Based on key epigenetic features identified in our RSF model, we will develop more accessible clinical detection methods (such as quantitative PCR or immunohistochemistry) to facilitate risk stratification in clinical practice.
4. **Drug Intervention Experiments**: Targeting the key epigenetic regulatory pathways we identified, we plan to conduct in vitro and in vivo drug intervention experiments to evaluate the potential for targeting these pathways, particularly for high-risk patients.
5. **Prospective Clinical Studies**: We intend to initiate prospective clinical studies to validate the value of the RSF model in guiding individualized treatment decisions for AML patients, assessing whether personalized treatment regimens based on RSF scores can improve therapeutic outcomes for patients with different risk profiles.

We believe these follow-up studies will not only validate our current computational findings but also further elucidate the relationship between epigenetic regulation and the immune microenvironment in AML, ultimately facilitating the development of individualized treatment strategies to improve patient outcomes.

We again thank the reviewer for their valuable comments, which will greatly help us refine our research and enhance its translational value.
